# Commissioning and performance evaluations of the electron Monte Carlo algorithm in a new commercial treatment planning system

**DOI:** 10.1002/acm2.70698

**Published:** 2026-07-17

**Authors:** Feng Wang, Xuming Jiang, Zhi Shi, Yanfang Liu, Yibin Zhang, Jiayi Chen

**Affiliations:** ^1^ Department of Radiation Oncology, Ruijin Hospital Shanghai Jiaotong University School of Medicine Shanghai China; ^2^ Shanghai Key Laboratory of Proton Therapy Shanghai China; ^3^ Institute for Medical Imaging Technology Shanghai China; ^4^ Shanghai United Imaging Healthcare Co. Shanghai China

**Keywords:** dose calculation accuracy, dose calculation time, electron monte carlo algorithm, and uTPS

## Abstract

**Purpose:**

This study provides a comprehensive evaluation of the newly implemented electron Monte Carlo dose calculation algorithm in the commercial treatment planning system, uTPS. It investigates the performance of the algorithm by examining its dosimetric accuracy and calculation speed, which have not been previously reported in the literature.

**Methods:**

To evaluate the dose calculation accuracy of the electron Monte Carlo algorithm in uTPS, the beam data from the electron energies of 6 MeV, 9 MeV, and 12 MeV in the newly developed dual‐layer MLC CT‐linac were modeled. Following AAPM MPPG 5.b, the calculated point/plane doses under normal and extended SSD, oblique incidence and cutouts, and heterogeneous phantoms with different energies were compared with the corresponding measurement data. The calculation time was evaluated by the comparisons between two different calculation workstations.

**Results:**

The beam data of 6 MeV, 9 MeV, and 12 MeV are well reproduced by the calculations. A dose grid resolution of 2 mm, 1% uncertainty, and dose to medium mode were used. Calculations successfully validated against the measured beam data, achieving mean dose differences of less than 2% in a water tank. The gamma passing rates were generally high and mostly exceeded 95% using 3 mm/3% criteria, although several larger‐field, extended‐SSD, and oblique/cutout cases showed lower passing rates, with minimum values remaining above 90%. The cutout tests, both special designed and patient specific, demonstrate that the algorithm can handle cases with cutouts effectively. In the heterogeneous phantom tests, the point dose differences for all SSDs are within 3.3%, indicating the accuracy of the uTPS Monte Carlo algorithm in handling heterogeneous media. The calculation times ranged from approximately 5 – 30 s for a statistical uncertainty of 1%, whereas achieving a tighter uncertainty of 0.5% increased the computation time to between 10 s and 2.5 min.

**Conclusion:**

Verifications performed under these basic and complicated conditions indicate that the accuracy of electron Monte Carlo algorithm in uTPS meets the requirements of MPPG 5.b for electron beam dose verification. The electron Monte Carlo algorithm of uTPS demonstrates accuracy comparable to that of other commercial TPSs. The comprehensive evaluation confirms the algorithm's robust performance, as it achieves the necessary levels of dosimetric precision and calculation times to support diverse radiotherapy workflows.

## 1 INTRODUCTION

Electron beam therapy, also known as electron radiotherapy, is a well‐established modality for treating superficial tumors, owing to its precise dose localization. It is frequently employed in head and neck cancers, chest wall irradiation for breast cancer, and the management of skin and lip cancers, as well as nodal boosts. The accuracy of dose calculation is of paramount importance in electron beam therapy, as it fundamentally underpins the delivery of the intended treatment. In radiotherapy, the Monte Carlo (MC) method is widely regarded as the gold standard for dose calculation, providing benchmark dose distributions that serve as a primary reference for various applications. MC methods for electron beam dose calculation have evolved from a benchmark of accuracy to a clinically implemented tool. Early research by Ma et al.^1^, ^2^ established the fundamental superiority of MC over pencil‐beam algorithms and developed accurate beam models. This foundation enabled subsequent work on clinical integration, focusing on the commissioning process^3^, ^4^ and the development of efficient verification techniques.^5^ Collectively, these efforts cemented the role of MC as a gold standard in treatment planning, as later reviewed by Reynaert et al.,^6^ underscoring its critical importance for precision radiotherapy. For the present, almost all the mainstream commercial treatment planning systems (TPSs) use a MC based algorithm in the electron dose calculation. Various works have been performed for the evaluation of the electron Monte Carlo calculations in these TPSs, for example Eclipse,^7^, ^8^, ^9^, ^10^, ^11^ Monaco,^12^ and RayStation.^13^

The uTPS is a new commercial treatment planning system compatible with photon and electron radiation therapy developed by the United Imaging Healthcare (UIH) Co., Ltd. While previous research on the uTPS platform and its predecessor uRT‐TPOIS has exclusively focused on photon therapy,^14^, ^15^, ^16^, ^17^, ^18^, ^19^, ^20^ the newly implemented electron Monte Carlo (eMC) algorithm remains uncharacterized. This study aims to fill this gap by conducting the first performance assessment of the newly implemented electron MC dose calculation capability in uTPS. This paper presents a comprehensive performance evaluation of the GPU‐accelerated eMC algorithm in the uTPS treatment planning system, in conjunction with a novel dual‐layer MLC CT‐linac. The assessment encompasses both dose calculation accuracy and computational speed.

The paper is organized as follows. In the section of materials and methods, an overview of the characteristics of the newly developed CT‐linac VisionaryTx together with the electron dose calculation algorithms in uTPS is provided. Then, under the guidance of AAPM MPPG 5.a^21^ and its latest version, MPPG 5.b,^22^ we detail the measurement equipment and procedures employed in this study. The results section presents a comparative analysis of measured and calculated doses, along with the corresponding computation times, for all applicators at electron energies of 6 MeV, 9 MeV, and 12 MeV. The discussions of dose calculation accuracy and calculation speed are presented in the following section. Finally, a short summary is given in the conclusion section.

## 2 MATERIALS AND METHODS

### 2.1 Specification of uLinac VisionaryTx

**FIGURE 1:**
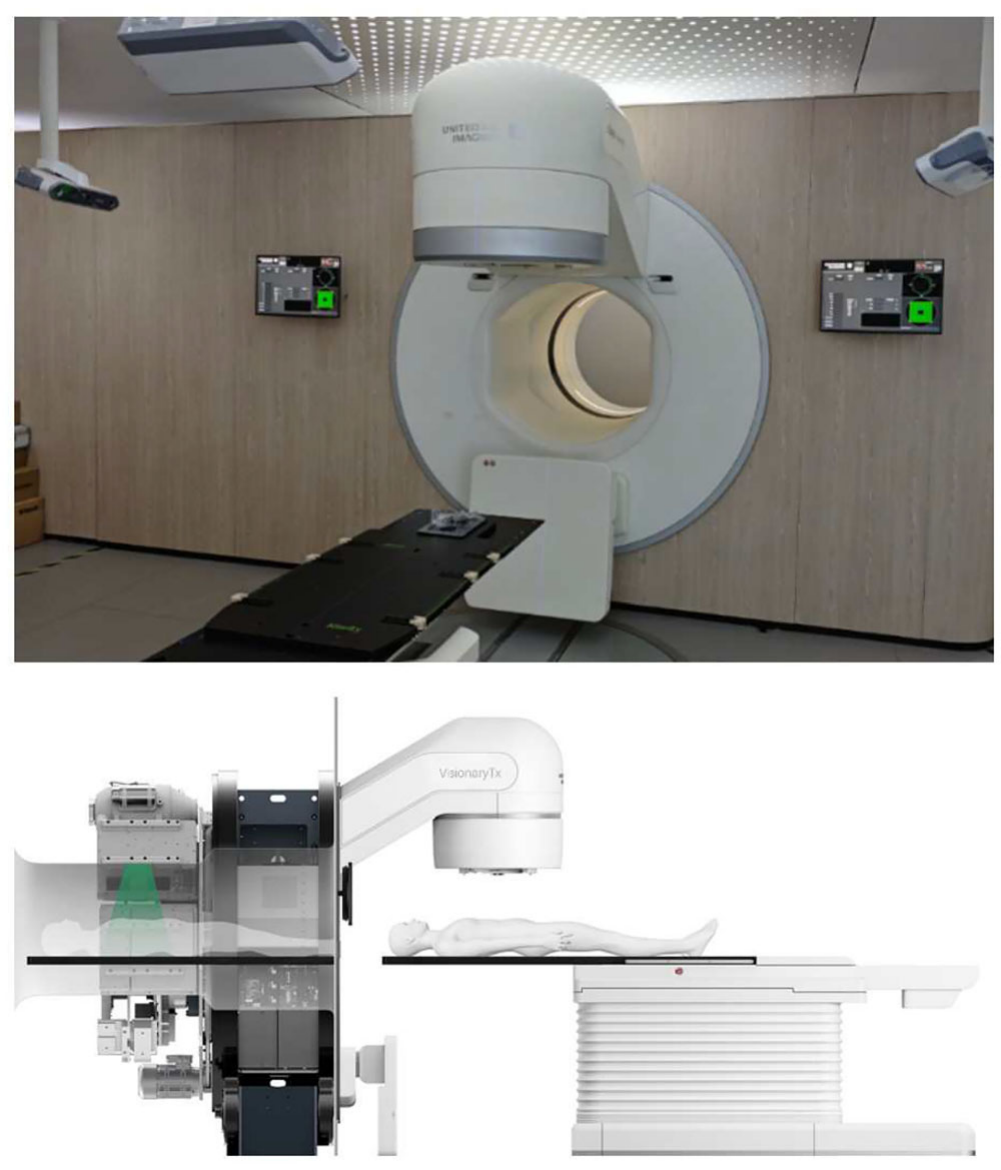
Photo and schematic view of the CT‐linac platform uLinac VisionaryTx.

**FIGURE 2:**
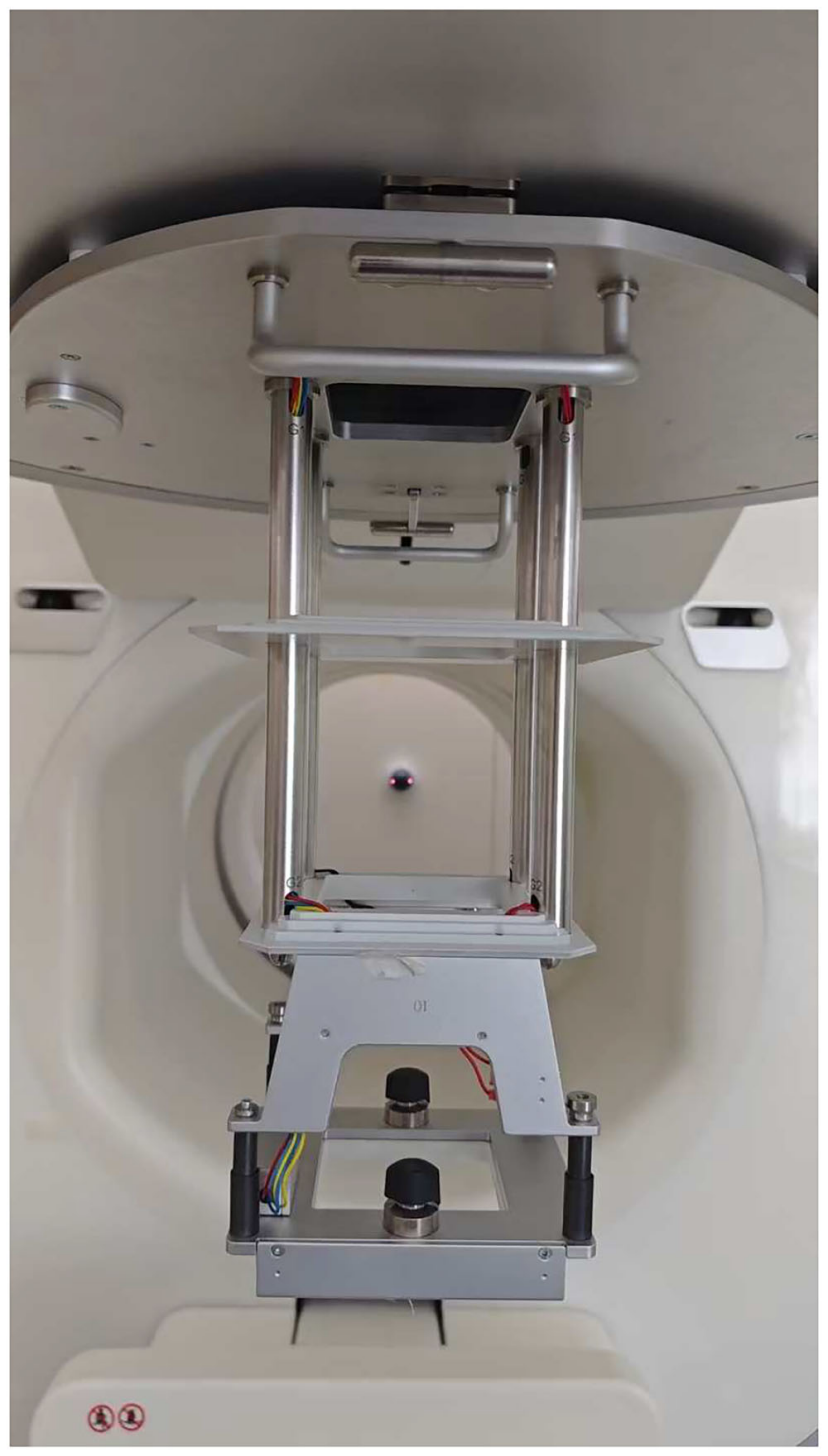
Photo of 10×10 ${\mathrm{cm}}^{2}$ applicator of uLinac VisionaryTx.

The CT‐integrated linac named uLinac VisionaryTx, as shown in Figure 1, along with the treatment planning system uTPS (version R001) both developed by UIH has been installed in Ruijin Hospital recently for the purpose of clinical trial.^23^ The CT‐linac is designed to deliver photon beams in 6 MV and 10 MV energies with flattened mode (with flattening filter, FF) and un‐flattened mode (flattening‐filter free, FFF) for both energies. An important feature of the beam‐limiting head is the equipped with 51 pairs of dual‐layered of MLCs results in the 2.5 mm width at the isocenter in the inner 10 cm and the 5 mm width in the outer 30 cm, allowing for a maximum projecting field size of 40×40 ${\mathrm{cm}}^{2}$. The CT‐linac system incorporates a 6 degree‐of‐freedom couch and a diagnostic‐quality 80‐slice helical CT, with 87 cm bore size and 63 cm standard field of view, and is particularly suitable for high‐precision treatment, for example SRS/SBRT and adaptive radiation therapy (ART). Four energies could be used in the electron radiation mode, i.e., 6 MeV, 9 MeV, 12 MeV, and 15 MeV. Five electron applicators could be used for each electron energy, that is, the applicators with sizes of 6×6 ${\mathrm{cm}}^{2}$, 10×10 ${\mathrm{cm}}^{2}$, 15×15 ${\mathrm{cm}}^{2}$, 20×20 ${\mathrm{cm}}^{2}$, and 25×25 ${\mathrm{cm}}^{2}$. The photo of 10×10 ${\mathrm{cm}}^{2}$ applicator of uLinac VisionaryTx is shown in Figure 2. The electron applicator has a four‐layer structure with a height of approximately 44 cm. Its main body is made of stainless steel, while the lower three collimator layers are constructed from aluminum‐iron alloy. At the bottom layer of the applicator, standard block or patient‐specific cutout can be placed. The lowest layer of electron applicator is placed at the position of the source‐to‐surface distance (SSD) equal to 95 cm.

### 2.2 Dose calculation algorithm in uTPS

The uTPS (version R001) is a commercial treatment planning system developed by UIH with browser/server (B/S) architecture, and can be used to formulate photon beam (three‐dimensional conformal, fixed field intensity‐modulated, volume intensity‐modulated, and rotational conformal) and electron beam external beam radiotherapy plans. The uTPS system employs two advanced photon dose calculation algorithms: Collapsed Cone Convolution Superposition (CC) and MC. To simulate the electron scatter precisely, a MC based electron dose calculation algorithm has been implemented in the uTPS for the electron radiation therapy.

The MC algorithm works by simulating the random behavior of particles many times. It then uses the statistical results from all these simulations to calculate a final, accurate answer.^24^, ^25^ The method obtains approximate solutions by using a computer to perform random sampling and statistical simulation based on a specific probability model. The MC dose calculation method solves the radiation transport problem by simulating the random histories of individual particles. At the beginning of the simulation, the initial particle information (including particle type, energy, position, and direction) is determined by sampling the beam model. Then, random sampling is used to determine the stochastic interactions between particles and medium during transport, track the resulting particle trajectories, and record the energy deposited in various voxels until the particle energy falls below a preset threshold. Additionally, the data from any secondary particles produced in these interactions are recorded and incorporated into the ongoing simulation. Finally, the energy deposited in various voxels is converted into absorbed dose. To simulate the electrons and photons out from the beam limiting device in the linac, including electron applicator, a virtual source model^26^, ^27^ is used in the electron MC algorithm to sample the energy, direction and velocity of each primary, secondary electron as well as contamination photon.

The (electron) MC algorithm in uTPS uses the CPU‐GPU asynchronous computing architecture to ensure the computational efficiency, that is, the CPU is used to handle the CT image and fluence map, and GPU is used to sample the particles emitted from the treatment head and simulate the particle transport in the patient. Due to the massive parallelization ability of graphics processing unit (GPU), thousands of times more floating‐point operations per second could be performed than with central processing unit (CPU). Therefore, to satisfy the clinical requirement of the calculation time, both the photon and electron dose calculation algorithms in uTPS are developed under the compute unified device architecture (CUDA) platform developed by NVIDIA. The computational performance was evaluated on two distinct workstations:

This configuration represents a trade‐off, where Type 1 features more powerful GPUs, whereas Type 2 is equipped with superior CPUs and RAM.

### 2.3 Dosimeters and phantoms

**FIGURE 3:**
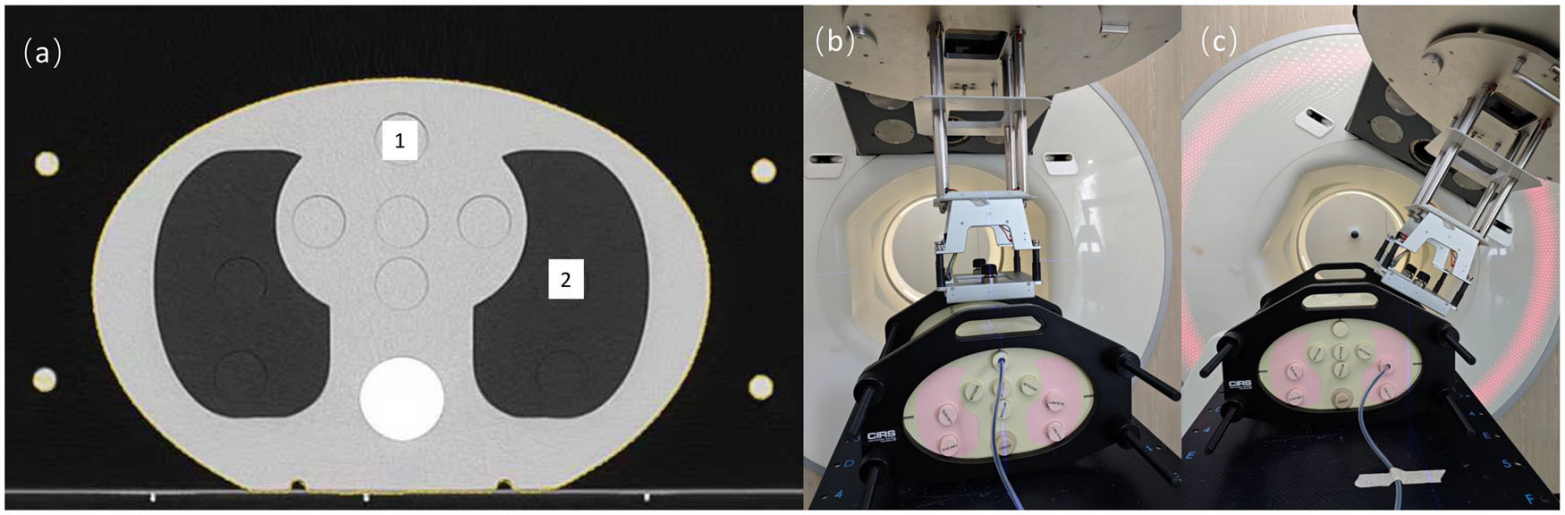
Measurement setup for the heterogeneous test. Point 1 is water equivalent, point 2 is lung substitutes.

For the collection of beam commissioning and validation data, an IBA Blue Phantom2 water tank (IBA Dosimetry GmbH, Germany) controlled by myQA‐Accept 8.5 software was employed. Two types of detectors were employed: a small‐sized cylindrical ion chamber (Scanditronix CC13, 0.13cc, IBA) for the measurement of percent depth dose (PDD) and profile curves under various measurement conditions and a 0.6cc Farmer chamber (PTW 30013, Freiburg, Germany) for the absolute dose calibration. For the plane dose verification, the 2D ion chamber array MatriXX (IBA Dosimetry GmbH, Germany) was used under different SSDs and water equivalent depths. The CIRS phantom (Model 002LFC) was used to verify the dose accuracy in heterogeneous material. The CIRS phantom can not only be used to verify the accuracy of the uTPS MC algorithm under conditions of heterogeneous media, but also for curved surfaces, and oblique incidence. The measurement setup is shown in Figure3. In the heterogeneous phantom, point 1 is water equivalent, while point 2 is lung substitutes. As shown in Figure3, point doses at point 1 and point 2 in the CIRS phantom were measured using a CC13 ion chamber for various energies and SSD values. A 10×10 ${\mathrm{cm}}^{2}$ electron applicator was selected. Since the minimum distance from the two measurement points to the phantom surface was greater than or equal to 3 cm, only 9 MeV and 12 MeV were measured to ensure a sufficient dose could be detected. In addition, the gantry angle was set to 30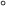 to ensure sufficient dose could be measured at the point 2 measurement point. For the patient‐specific quality assurance (PSQA) of clinical case, an IBA slab phantom in water‐equivalent RW3 material with dimensions of 30 cm×30 cm×10 cm and the CC13 ion chamber were used for the measurement of absolute point dose verification.

### 2.4 Beam modeling

To commission the eMC algorithm, its parameters were adjusted against measured beam data from the CT‐Linac. Following the manufacturer's suggestions, the beam data were acquired at an SSD of 100 cm for energies of 6, 9, and 12 MeV with all applicators:

- Percentage Depth Dose (PDD)
- Crossline profiles at depth of dmax (positions of max dose), R80 (80% of the max dose), and R30 (30% of the max dose) in water
- Output factors

The 15 MeV electron energy was not available for this study. The complete set of beam data required for algorithm commissioning is listed in Table 2. The output factors were measured at the position of max dose for each energy with SSD equal to 100 cm (Table 1).

**TABLE 1:** Comparisons of the two types of workstations.

	type1	type2	comparison
GPU	2 ×NVIDIA RTX 2080 (8 GB each)	2 ×NVIDIA RTX 2060 Super (8 GB each)	Type1 better
CPU	2 ×Intel Xeon E5‐2630v2	2 ×Intel Xeon W‐2245 CPUs	Type2 better
Memory	64 GB DDR3 RAM	64 GB DDR4 RAM	Type2 better

**TABLE 2:** Required beam data for 6 MeV, 9 MeV, and 12 MeV with all applicator sizes.

Energy	Data type	Measure depth (cm)
6 MeV	PDD	—
	Profile	1.3, 2.0, 3.0
	Output factor	1.3
9 MeV	PDD	—
	Profile	2.0, 3.0, 4.0
	Output factor	2.0
12 MeV	PDD	—
	Profile	2.6, 4.0, 5.0
	Output factor	2.6

During beam data acquisition, precise detector positioning on the central axis was achieved by performing system alignment based on the lateral profiles acquired along the principal axes at each measurement depth to reduce the measurement uncertainty introduced by water tank positioning. The absolute doses at the positions of max doses were calibrated to be 100 cGy with SSD equal 100 cm and 100 MU under the applicator size of 10×10 ${\mathrm{cm}}^{2}$ for each energy. The positions of max dose in the water were depths 1.3 cm, 2.0 cm, and 2.6 cm under the condition of SSD equal to 100 cm with 10×10 ${\mathrm{cm}}^{2}$ applicator for the energies of 6 MeV, 9 MeV, and 12 MeV, respectively.

During electron beam modeling process, the beam data obtained under the above measurement conditions were imported to the modeling module in uTPS. Before importing into the modeling module in uTPS, the CAX corrections and symmetrizations were performed for the measured profile curves in the myQA‐Accept software. The PDD from 10×10 ${\mathrm{cm}}^{2}$ applicator of each energy was used to fit the energy spectrum parameters automatically, including the primary electrons, secondary electrons and contamination photons. The PDDs from other applicators were used to verify the obtained energy spectrum. Then the profiles from the maximum size, that is, 25×25 ${\mathrm{cm}}^{2}$ applicator of each energy were used to perform the off‐axis correction. Finally, the measured output factor from each applicator was used to correct the calculated output factor.

### 2.5 Evaluation of dose calculation accuracy

Following the recommendations for electron dose in AAPM MPPG 5.b,^22^ the dose calculation accuracies were evaluated by the comparisons of the measurements and calculations, including tests with normal (SSD 100 cm) and extended SSDs (SSD 105 cm and 110 cm), oblique incidence, irregular cutouts and heterogeneous phantom. After the basic validations were completed, in all the calculations, the grid resolution was all set as 2 mm, the calculation uncertainty was set as 0.5% for point dose verifications to make the dose fluctuations lower and 1% for plan dose and clinical case verifications to simulate the clinical scenarios. In the uTPS, options for dose to water and dose to medium are also provided; in this study, dose to medium was selected for all calculations.

In order to evaluate the accuracy of the dose calculation algorithm more comprehensively, the point doses were measured in the IBA blue phantom2 water tank with SSD 100 cm, 105 cm, and 110 cm. The depths of the measurements were the same as those of commissioning beam data. For each depth, three positions were selected, including central axis, the flatten area, and the penumbra low dose region. For each point, 200 MU were delivered with SSDs equal to 100 cm, 105 cm, and 110 cm. Since the MC algorithm is a method based on probability and statistics theory, an important feature of dose distribution calculated by MC algorithm is the fluctuations in the dose distribution. So a single point may be located at the peak or valley of the fluctuation, this could overestimate or underestimate the dose difference. As recommended in AAPM TG105,^28^ it is usual to score quantities (dose mainly) averaged over some finite volume or voxel. Therefore, a region of interest (ROI) of the detector was contoured. The center of the ROI was located at the corresponding measured position, and the size of the ROI was equal to the effective detector size of the ion chamber. The mean value in this ROI was regarded as the point dose at the corresponding position.

Following the MPPG 5.b's recommendation, the dose differences percentage for the points are of the central ray normalization dose. Therefore, the dose difference for normal and extended SSDs from the water tank measurements were calculated using the following Equation 1,

(1) $$\mathrm{Diff}.=\frac{D_{\mathrm{mea}.}-D_{\mathrm{calc}}}{D_{0}}$$

Here, $D_{\mathrm{mea}.}$ and $D_{\mathrm{calc}}$ denote the doses from measurements and calculations at the same position, $D_{0}$ means the measured dose from the central axis position with the same depth of $D_{\mathrm{mea}.}$

For the relative point doses difference from CIRS heterogeneous phantom and solid water phantom, the Equation 2 is used.

(2) $$\mathrm{Diff}.=\frac{D_{\mathrm{mea}.}-D_{\mathrm{calc}}}{D_{\mathrm{mea}.}}$$

For the plane dose verification, the gamma criterion of 3%/3 mm was used. The dose threshold was set to 10%, and the global and absolute modes were selected.

## 3 RESULTS

This section presents a comprehensive set of results evaluating the electron dose calculation accuracy under the suggestions of MPPG 5.b and computational speed of the algorithm. The analysis encompasses all available applicators across 6, 9, and 12 MeV electron energies under various test conditions.

### 3.1 Beam modeling results

**FIGURE 4:**
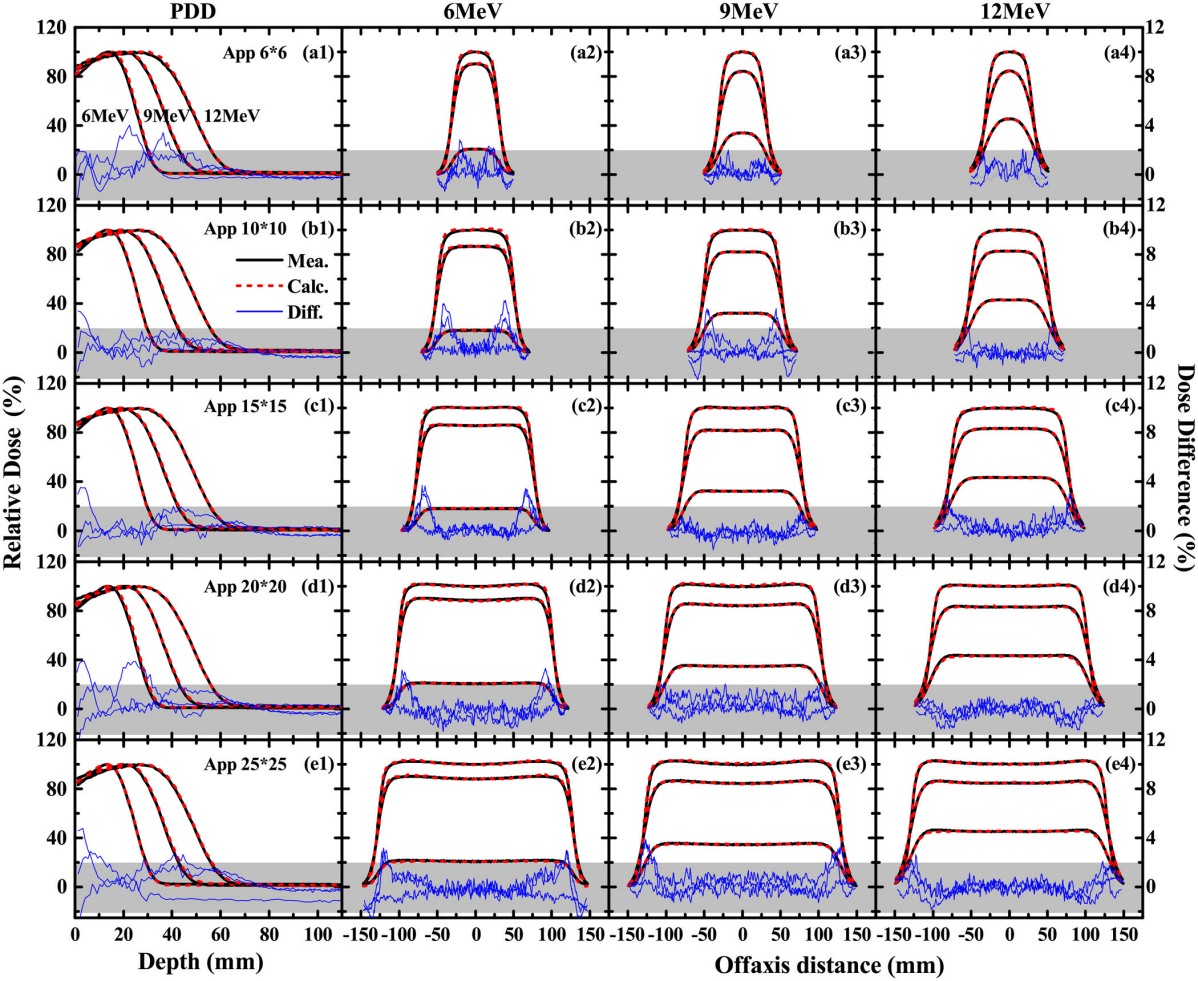
(a1)–(e1): The comparisons of measured and calculated PDD curves for 6 MeV, 9 MeV, and 12 MeV from 6×6 ${\mathrm{cm}}^{2}$ to 25×25 ${\mathrm{cm}}^{2}$ applicators. (a2)–(e2):The comparisons of measured and calculated profiles around the depths of $d_{\max}$, R80, and R30 for all applicators of 6 MeV. (a3)–(e3): Same as (a2)–(e2) but for 9 MeV. (a4)–(e4): Same as (a2)–(e2) but for 12 MeV.The black solid lines represent the measurement data, the red short dash lines represent the calculation values, and the blue lines represent the differences between the calculations and measurements of PDDs and profiles. The shaded areas represent the region within a relative difference of 2% between the calculations and measurements.

The final beam modeling results are shown in Figure 4 and Table 3. In Figure 4, the subfigures (a1)– (e1) correspond to the comparisons of the PDD curves for the energies of 6 MeV, 9 MeV, and 12 MeV from the smallest to the largest applicators, that is, from 6×6 ${\mathrm{cm}}^{2}$ to 25×25 ${\mathrm{cm}}^{2}$ applicators. The subfigures (a2)– (e2), (a3)–(e3), and (a4)–(e4) represent the comparisons of the profiles around the depths of max dose, R80, and R30 for all five applicators of 6 MeV, 9 MeV, and 12 MeV, respectively. The black solid lines represent the measurement data, the red short dash lines represent the calculations, and the blue solid lines represent the differences between the calculations and measurements. The shaded areas represent the region within a relative dose difference of 2%.

**TABLE 3:** One dimension gamma passing rates with the criteria of 2 mm, 2% for the PDDs and profiles at different depth for the energies of 6 MeV, 9 MeV, and 12 MeV with all applicators. For PDDs (excluding the buildup region), the distance to agreement (DTA) analysis with the criteria of 3 mm were also used.

Energy	Curve type	Depth	Analysis method	6×6 ${\mathrm{cm}}^{2}$	10×10 ${\mathrm{cm}}^{2}$	15×15 ${\mathrm{cm}}^{2}$	20×20 ${\mathrm{cm}}^{2}$	25×25 ${\mathrm{cm}}^{2}$
6 MeV	PDD	—	DTA	100.00%	100.00%	100.00%	100.00%	100%
		—	γ	100.00%	96.77%	96.77%	96.77%	96.77%
	Profile	1.3 cm	γ	100.00%	84.03%	94.38%	95.05%	100.00%
		2.0 cm	γ	100.00%	98.78%	99.43%	100.00%	100.00%
		3.0 cm	γ	100.00%	100.00%	100.00%	100.00%	100.00%
9 MeV	PDD	—	DTA	100.00%	100.00%	100.00%	100.00%	100.00%
		—	γ	100.00%	100.00%	100.00%	100.00%	100.00%
	Profile	2.0 cm	γ	100.00%	100.00%	100.00%	100.00%	100.00%
		3.0 cm	γ	100.00%	100.00%	100.00%	100.00%	100.00%
		4.0 cm	γ	100.00%	100.00%	100.00%	100.00%	100.00%
12 MeV	PDD	—	DTA	100.00%	100.00%	100.00%	100.00%	100.00%
		—	γ	100.00%	100.00%	100.00%	98.31%	100.00%
	Profile	2.6 cm	γ	100.00%	100.00%	100.00%	100.00%	100.00%
		4.0 cm	γ	100.00%	100.00%	100.00%	100.00%	100.00%
		5.0 cm	γ	100.00%	100.00%	100.00%	100.00%	100.00%

From Figure 4, it is clearly seen that the agreement between the measurements and calculations is quite good for all curves, including the high dose and penumbra regions in the profiles and build up and fall off regions as well as the contamination photon tails in the PDDs. Most of the dose differences, as shown with the blue solid lines, are concentrated on the shaded areas, that is, within 2%. For PDDs, the dose differences are a bit larger for 6 MeV with 6×6 ${\mathrm{cm}}^{2}$ and 20×20 ${\mathrm{cm}}^{2}$. The maximum dose differences reach around 4% for both situations. For profiles, the dose differences for all the three energies mainly come from the penumbra regions, and the dose differences of 6 MeV with 10×10 ${\mathrm{cm}}^{2}$ applicators are larger than others. The poorer agreement for 6 MeV compared with 9 MeV and 12 MeV mainly because that 6 MeV electron generate more low‐energy scattering from the treatment head and applicator than 9 MeV and 12 MeV which make the situation more complicated to model the beam data.

Besides the comparisons of the measured and calculated curves shown in Figure 4, the one dimensional gamma passing rates (GPRs) with the criteria of 2 mm, 2% were also used to evaluate beam modeling from another perspective. As illustrated in Table 3, for the results from 9 MeV and 12 MeV, nearly all the GPRs are reaching 100%, except the GPR of 12 MeV PDD with 20×20 ${\mathrm{cm}}^{2}$ applicator. While the GPRs of 6 MeV are a little worse than those of 9 MeV and 12 MeV, especially for the results of profiles at the depth of 1.3 cm. As suggested in MPPG 5.b, for PDDs (excluding the buildup region), the distance to agreement (DTA) analysis with the criteria of 3 mm were also used. The DTA passing rates were 100% for all the PDDs.

### 3.2 Point dose verification

The dose differences for normal SSD 100 cm and extended SSDs 105 cm and 110 cm are listed in Tables 4, 5, and 6 for 6 MeV, 9 MeV, and 12 MeV, respectively. The detailed measured and calculated absolute doses as well as dose differences for 6 MeV, 9 MeV, and 12 MeV could be found in the supplementary data.

**TABLE 4:** The dose difference (Diff.) between measurements and calculations for the energy of 6 MeV at different positions under 200 MU and SSDs 100 cm, 105 cm, and 110 cm.

6×6 ${\mathrm{cm}}^{2}$ applicator									
Off axis distance	SSD=100 cm Diff.			SSD=105 cm Diff.			SSD=110 cm Diff.		
	0.0 cm	1.5 cm	4.0 cm	0.0 cm	1.5 cm	4.0 cm	0.0 cm	1.5 cm	4.0 cm
depth 1.3 cm	0.00%	−1.97%	0.86%	−2.36%	−2.80%	1.84%	−0.99%	−2.65%	2.77%
depth 2.0 cm	−2.61%	−3.55%	0.60%	0.07%	−1.11%	3.48%	−3.35%	−4.80%	5.31%
depth 3.0 cm	2.69%	1.94%	−0.38%	5.86%	4.92%	5.83%	−3.85%	−5.72%	4.94%
10×10 ${\mathrm{cm}}^{2}$ applicator									
Off axis distance	SSD=100 cm Diff.			SSD=105 cm Diff.			SSD=110 cm Diff.		
	0.0 cm	2.5 cm	6.0 cm	0.0 cm	2.5 cm	6.0 cm	0.0 cm	2.5 cm	6.0 cm
depth 1.3 cm	−0.29%	−1.81%	0.39%	−1.91%	−2.52%	4.09%	−2.31%	−3.43%	2.04%
depth 2.0 cm	−0.90%	−0.94%	0.81%	−1.58%	−1.52%	2.52%	−3.29%	−2.95%	3.67%
depth 3.0 cm	0.03%	−1.84%	−0.86%	4.97%	0.36%	0.89%	−4.70%	−4.92%	5.20%
15×15 ${\mathrm{cm}}^{2}$ applicator									
Off axis distance	SSD=100 cm Diff.			SSD=105 cm Diff.			SSD=110 cm Diff.		
	0.0 cm	3.0 cm	9.0 cm	0.0 cm	3.0 cm	9.0 cm	0.0 cm	3.0 cm	9.0 cm
depth 1.3 cm	0.59%	0.77%	0.13%	0.54%	0.75%	3.92%	0.85%	0.87%	3.75%
depth 2.0 cm	−0.87%	−1.49%	0.31%	2.91%	2.77%	3.63%	0.00%	1.03%	5.88%
depth 3.0 cm	−0.70%	0.00%	−1.83%	5.63%	1.57%	0.07%	−5.68%	−4.54%	1.32%
20×20 ${\mathrm{cm}}^{2}$ applicator									
Off axis distance	SSD=100 cm Diff.			SSD=105 cm Diff.			SSD=110 cm Diff.		
	0.0 cm	5.0 cm	12.0 cm	0.0 cm	5.0 cm	12.0 cm	0.0 cm	5.0 cm	12.0 cm
depth 1.3 cm	0.45%	1.48%	−0.43%	1.76%	2.07%	2.79%	0.64%	1.51%	4.30%
depth 2.0 cm	−0.75%	−0.59%	−0.11%	3.06%	3.98%	3.45%	−0.76%	−0.37%	5.88%
depth 3.0 cm	−0.29%	−1.79%	−0.89%	1.93%	1.62%	−0.38%	−5.62%	−4.31%	0.85%
25×25 ${\mathrm{cm}}^{2}$ applicator									
Off axis distance	SSD=100 cm Diff.			SSD=105 cm Diff.			SSD=110 cm Diff.		
	0.0 cm	7.0 cm	14.0 cm	0.0 cm	7.0 cm	14.0 cm	0.0 cm	7.0 cm	14.0 cm
depth 1.3 cm	0.28%	−1.22%	0.98%	−0.79%	−0.84%	3.87%	−0.81%	−1.22%	0.72%
depth 2.0 cm	−1.59%	−1.60%	0.10%	0.99%	1.15%	3.70%	−2.79%	−1.83%	−1.00%
depth 3.0 cm	0.30%	2.44%	1.71%	3.64%	3.42%	0.86%	−3.38%	0.63%	−5.51%

**TABLE 5:** Same as Table 4, but for the energy of 9 MeV.

6×6 ${\mathrm{cm}}^{2}$ applicator									
Off axis distance	SSD=100 cm Diff.			SSD=105 cm Diff.			SSD=110 cm Diff.		
	0.0 cm	1.5 cm	4.0 cm	0.0 cm	1.5 cm	4.0 cm	0.0 cm	1.5 cm	4.0 cm
depth 2.0 cm	−0.15%	−0.86%	0.08%	−1.43%	−2.76%	2.76%	−2.65%	−2.17%	1.27%
depth 3.0 cm	−0.29%	−0.57%	0.29%	−0.29%	−1.45%	2.28%	−2.33%	−3.83%	3.44%
depth 4.0 cm	1.76%	0.73%	−0.67%	0.99%	0.31%	0.56%	−1.06%	−1.91%	1.99%
10×10 ${\mathrm{cm}}^{2}$ applicator									
Off axis distance	SSD=100 cm Diff.			SSD=105 cm Diff.			SSD=110 cm Diff.		
	0.0 cm	2.5 cm	6.0 cm	0.0 cm	2.5 cm	6.0 cm	0.0 cm	2.5 cm	6.0 cm
depth 2.0 cm	−0.51%	−0.61%	0.84%	0.88%	−0.62%	3.90%	−1.90%	−1.72%	1.51%
depth 3.0 cm	−0.51%	−0.40%	0.72%	−1.09%	−1.57%	1.50%	−2.03%	−2.34%	1.82%
depth 4.0 cm	0.52%	0.88%	−0.48%	−2.85%	−1.98%	−1.25%	−2.17%	−3.26%	−2.54%
15×15 ${\mathrm{cm}}^{2}$ applicator									
Off axis distance	SSD=100 cm Diff.			SSD=105 cm Diff.			SSD=110 cm Diff.		
	0.0 cm	3.0 cm	9.0 cm	0.0 cm	3.0 cm	9.0 cm	0.0 cm	3.0 cm	9.0 cm
depth 2.0 cm	0.02%	−0.04%	0.42%	1.22%	1.73%	3.72%	1.25%	0.97%	2.81%
depth 3.0 cm	−0.49%	0.01%	−0.13%	2.37%	2.54%	2.72%	1.71%	2.56%	2.68%
depth 4.0 cm	−0.64%	−0.05%	−1.61%	−1.69%	−1.11%	−0.51%	−3.33%	−2.49%	0.69%
20×20 ${\mathrm{cm}}^{2}$ applicator									
Off axis distance	SSD=100 cm Diff.			SSD=105 cm Diff.			SSD=110 cm Diff.		
	0.0 cm	5.0 cm	12.0 cm	0.0 cm	5.0 cm	12.0 cm	0.0 cm	5.0 cm	12.0 cm
depth 2.0 cm	0.19%	−0.71%	0.03%	1.44%	1.62%	2.85%	1.44%	0.50%	2.38%
depth 3.0 cm	0.93%	1.44%	−0.25%	1.30%	0.69%	1.57%	0.72%	1.22%	3.47%
depth 4.0 cm	0.41%	1.56%	−1.61%	−3.19%	−2.21%	−1.63%	−3.71%	−3.91%	−0.60%
25×25 ${\mathrm{cm}}^{2}$ applicator									
Off axis distance	SSD=100 cm Diff.			SSD=105 cm Diff.			SSD=110 cm Diff.		
	0.0 cm	7.0 cm	14.0 cm	0.0 cm	7.0 cm	14.0 cm	0.0 cm	7.0 cm	14.0 cm
depth 2.0 cm	0.45%	−0.94%	−0.41%	0.67%	1.48%	2.60%	0.27%	0.70%	−0.39%
depth 3.0 cm	1.37%	1.42%	−1.00%	2.67%	3.18%	2.06%	2.13%	3.62%	0.85%
depth 4.0 cm	1.95%	2.04%	−1.18%	0.69%	3.42%	−0.04%	−1.53%	3.07%	1.59%

**TABLE 6:** Same as Table 4, but for the energy of 12 MeV.

6×6 ${\mathrm{cm}}^{2}$ applicator									
Off axis distance	SSD=100 cm Diff.			SSD=105 cm Diff.			SSD=110 cm Diff.		
	0.0 cm	1.5 cm	4.0 cm	0.0 cm	1.5 cm	4.0 cm	0.0 cm	1.5 cm	4.0 cm
depth 2.6 cm	0.31%	0.01%	−0.93%	−1.88%	−2.98%	−0.83%	−2.42%	−2.34%	2.07%
depth 4.0 cm	−0.57%	−0.35%	−0.10%	0.34%	−0.22%	0.71%	−0.77%	−0.22%	0.62%
depth 5.0 cm	1.78%	2.66%	0.40%	2.32%	1.72%	2.00%	−0.10%	−1.03%	0.48%
10×10 ${\mathrm{cm}}^{2}$ applicator									
Off axis distance	SSD=100 cm Diff.			SSD=105 cm Diff.			SSD=110 cm Diff.		
	0.0 cm	2.5 cm	6.0 cm	0.0 cm	2.5 cm	6.0 cm	0.0 cm	2.5 cm	6.0 cm
depth 2.6 cm	0.35%	0.40%	−0.34%	0.21%	−0.42%	0.19%	−1.19%	−1.11%	2.47%
depth 4.0 cm	0.00%	0.30%	0.32%	1.79%	1.30%	0.79%	−0.51%	−1.31%	0.56%
depth 5.0 cm	0.22%	2.93%	1.34%	3.05%	3.28%	1.18%	−1.34%	−2.67%	−1.11%
15×15 ${\mathrm{cm}}^{2}$ applicator									
Off axis distance	SSD=100 cm Diff.			SSD=105 cm Diff.			SSD=110 cm Diff.		
	0.0 cm	3.0 cm	9.0 cm	0.0 cm	3.0 cm	9.0 cm	0.0 cm	3.0 cm	9.0 cm
depth 2.6 cm	0.58%	−0.29%	−1.06%	−0.04%	−0.37%	−0.66%	−0.09%	−0.61%	2.40%
depth 4.0 cm	−0.28%	−0.26%	−0.98%	2.96%	2.81%	0.50%	−0.31%	−0.05%	0.74%
depth 5.0 cm	−0.72%	−1.05%	−0.89%	3.34%	3.30%	1.98%	0.08%	0.36%	−0.35%
20×20 ${\mathrm{cm}}^{2}$ applicator									
Off axis distance	SSD=100 cm Diff.			SSD=105 cm Diff.			SSD=110 cm Diff.		
	0.0 cm	5.0 cm	12.0 cm	0.0 cm	5.0 cm	12.0 cm	0.0 cm	5.0 cm	12.0 cm
depth 2.6 cm	0.28%	−1.56%	−0.05%	−1.00%	−0.49%	0.01%	−0.40%	−0.70%	2.41%
depth 4.0 cm	−0.48%	−0.95%	−0.06%	0.62%	1.29%	0.17%	−1.45%	−2.07%	0.19%
depth 5.0 cm	−0.23%	0.46%	−0.21%	3.06%	3.53%	0.39%	−2.52%	−1.90%	−0.79%
25×25 ${\mathrm{cm}}^{2}$ applicator									
Off axis distance	SSD=100 cm Diff.			SSD=105 cm Diff.			SSD=110 cm Diff.		
	0.0 cm	7.0 cm	14.0 cm	0.0 cm	7.0 cm	14.0 cm	0.0 cm	7.0 cm	14.0 cm
depth 2.6 cm	−0.10%	−0.67%	−1.40%	−1.69%	−1.95%	−1.24%	−1.46%	−1.67%	−1.69%
depth 4.0 cm	0.09%	0.69%	−0.63%	2.80%	2.45%	−0.88%	−1.05%	−0.02%	−0.45%
depth 5.0 cm	2.17%	1.57%	0.69%	2.89%	2.23%	1.86%	1.24%	1.83%	−0.31%

From Tables 4, 5, and 6, it can be seen that the measured absolute dose under SSD 100 cm are well reproduced by the calculations. As shown in Table 4, for 6 MeV, nearly all the dose differences under SSD 100 cm are less than ±3%, except the result at the position of depth 2.0 cm with off axis distance 1.5 cm under 6×6 ${\mathrm{cm}}^{2}$, at which the dose difference is ‐3.55%. However, with increasing SSD, the dose deviation increased significantly. For 9 MeV and 12 MeV as shown in Tables 5 and 6, the dose differences of all the positions under SSD 100 cm are within ±3%. The maximum dose differences are 2.03% and 2.98% for 9 MeV and 12 MeV respectively. As the SSD increases, the dose deviations for both 9 MeV and 12 MeV increase, but both of them are smaller than those for 6 MeV. Furthermore, the dose variation for 12 MeV at different SSDs is not much significant.

### 3.3 Plane dose verification

Following the point dose verifications, the plane doses were also verified with IBA MatriXX under SSDs 100 cm, 105 cm, and 110 cm. During the MatriXX measurement, 5 cm thick solid water was placed under the MatriXX device to account for backscatter. The corresponding results are shown in Table 7. The depth in the second column of Table 7 means the thickness of the water equivalent slabs (including the buildup of MatriXX itself) above the detectors. The SSDs are listed in the third column. The GPRs are analyzed by the myQA‐Patient software with the criteria of 3 mm, 3% and 10% dose threshold.

**TABLE 7:** The global absolute GPRs with criteria of 3mm, 3% and 10% dose threshold for 6 MeV, 9 MeV, and 12 MeV with all applicators under SSDs 100 cm, 105 cm, and 110 cm.

Energy	Depth (cm)	SSD (cm)	6×6 ${\mathrm{cm}}^{2}$	10×10 ${\mathrm{cm}}^{2}$	15×15 ${\mathrm{cm}}^{2}$	20×20 ${\mathrm{cm}}^{2}$	25×25 ${\mathrm{cm}}^{2}$
6 MeV	1.3	100	100.0%	99.6%	100.0%	100.0%	99.1%
	1.3	105	100.0%	93.0%	98.4%	96.8%	96.8%
	1.3	110	99.4%	100.0%	99.0%	99.6%	90.8%
	2.0	100	100.0%	100.0%	100.0%	100.0%	97.8%
9 MeV	2.0	100	100.0%	100.0%	100.0%	100.0%	93.2%
	2.0	105	99.2%	99.7%	100.0%	100.0%	92.2%
	2.0	110	99.2%	100.0%	99.5%	98.9%	92.1%
	3.0	100	100.0%	100.0%	100.0%	100.0%	93.2%
12 MeV	2.6	100	100.0%	100.0%	100.0%	100.0%	93.1%
	2.6	105	100.0%	100.0%	99.8%	100.0%	92.0%
	2.6	110	100.0%	99.6%	100.0%	99.5%	95.5%
	4.0	100	100.0%	100.0%	100.0%	100.0%	93.3%

In Table 7, it is shown that all the GPRs were greater than 90% with the criteria of 3 mm, 3%. However, the GPRs of 25×25 ${\mathrm{cm}}^{2}$ for all the energies are much worse than other applicators. The average GPR for 25×25 ${\mathrm{cm}}^{2}$ was only 94.1%, while for all the other applicators, the average value was 99.6%.

### 3.4 Oblique incidence and cutout

**FIGURE 5:**
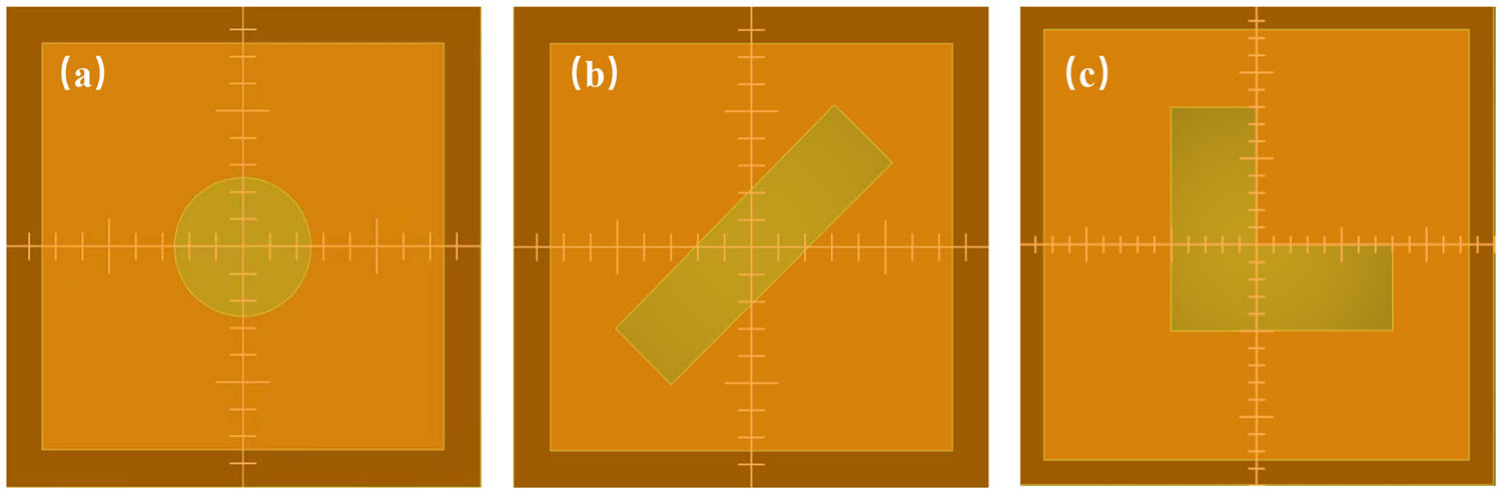
Schematic diagrams of the special designed cutouts from the beam eye view. Each tick in the figure represents 1 cm.

In the former test cases under normal and extended SSDs, the gantry angles was set to 0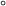, and no cutouts were used in the electron applicator. Therefore, to verify the accuracy of the algorithm under oblique incidence conditions, we selected the 10×10 ${\mathrm{cm}}^{2}$ applicator as reference applicator, and set the gantry angle to 30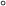. In addition, we tested the dose accuracy of the algorithm using cutouts of three different shapes. According to ECWG^29^ and AAPM TG53,^30^ three cutouts were selected. The three shapes consist of a circle with a diameter of 5 cm insert of 15×15 ${\mathrm{cm}}^{2}$ applicator, a 3×12 ${\mathrm{cm}}^{2}$ insert along the diagonal of 15×15 ${\mathrm{cm}}^{2}$ applicator, and an L‐shape insert of 25×25 ${\mathrm{cm}}^{2}$ applicator. The shapes of the three cutouts from the beam eye view are shown in the Figure5. Each tick in the figure represents 1 cm. The measured MatriXX doses at the positions of maximum dose for 6 MeV, 9 MeV, and 12 MeV at SSD of 100 cm, 105 cm, and 110 cm.

Plane doses for oblique incidence and the three cutouts were measured using a MatriXX detector at 6 MeV, 9 MeV, and 12 MeV under SSDs 100 cm, 105 cm, and 110 cm. Then, the measured plane doses were compared with the calculated results. The plane dose passing rates for the oblique and cutout test cases are shown in Table 8.

**TABLE 8:** The global absolute GPRs with criteria of 3mm, 3% and 10% dose threshold for 6 MeV, 9 MeV, and 12 MeV under oblique incidence and special cutout conditions with SSDs 100 cm, 105 cm, and 110 cm.

		10×10 ${\mathrm{cm}}^{2}$ applicator	15×15 ${\mathrm{cm}}^{2}$ applicator		25×25 ${\mathrm{cm}}^{2}$ applicator
Depth (cm)	SSD (cm)	Oblique	5 cm Circle	3×12 ${\mathrm{cm}}^{2}$ diagonal	L‐Shape
1.3	100	98.2%	100.0%	100.0%	99.6%
1.3	105	99.6%	100.0%	100.0%	100.0%
1.3	110	100.0%	100.0%	100.0%	100.0%
2	100	92.3%	100.0%	97.6%	99.2%
2	105	96.9%	100.0%	100.0%	100.0%
2	110	100.0%	100.0%	100.0%	100.0%
2.6	100	97.8%	96.2%	96.5%	92.6%
2.6	105	99.1%	97.5%	98.8%	99.1%
2.6	110	99.7%	100.0%	100.0%	100.0%

As can be seen from Table 8, except for the case of 9 MeV, SSD 100 cm under oblique incidence and 12 MeV, SSD 100 cm with L‐shaped cutout, all other gamma passing rates based on the 3%/3 mm criterion are greater than 95%.This demonstrates that the electron MC algorithm in uTPS shows good accuracy under oblique incidence and various cutout shapes at different SSD distances.

### 3.5 Heterogeneous phantom

The detailed measurement conditions, as well as the comparison between the measured and calculated results, are shown in the Table 9. The relative dose differences were calculated using Equation2. During the calculation, the effective volume of the detector in the CT images was contoured and assigned to water material.

**TABLE 9:** The measured and calculated point doses as well as relative dose difference for point 1 and point 2 in CIRS phantom.

Measurement position	SSD (cm)	Energy	Mea. (cGy)	Cal. (cGy)	Diff.
point 1	100	9 MeV	161.22	159.22	1.2%
		12 MeV	197.92	196.3	0.8%
	105	9 MeV	148.97	151.47	−1.7%
		12 MeV	178.30	178.17	0.1%
	110	9 MeV	134.78	137.72	−2.2%
		12 MeV	163.07	164.44	−0.8%
point 2	100	9 MeV	132.78	137.06	−3.2%
		12 MeV	177.6	173.26	2.4%
	105	9 MeV	123.72	127.75	−3.3%
		12 MeV	161.88	159.26	1.6%
	110	9 MeV	110.52	109.98	0.5%
		12 MeV	148.2	147.8	0.3%

It can be seen from the results in Table 9 that for point 1 (water‐equivalent material), all relative dose deviations are less than 3%, with a maximum dose deviation of ‐2.2%. For 9 MeV, the dose deviation gradually increases with varying SSD, while for 12 MeV, no obvious increasing trend in dose deviation is observed. For point 2 (lung‐substitute material), the maximum dose deviation is ‐3.3%. The dose deviations at point 2 are generally larger than those at point 1.

### 3.6 Clinical case verification

**FIGURE 6:**
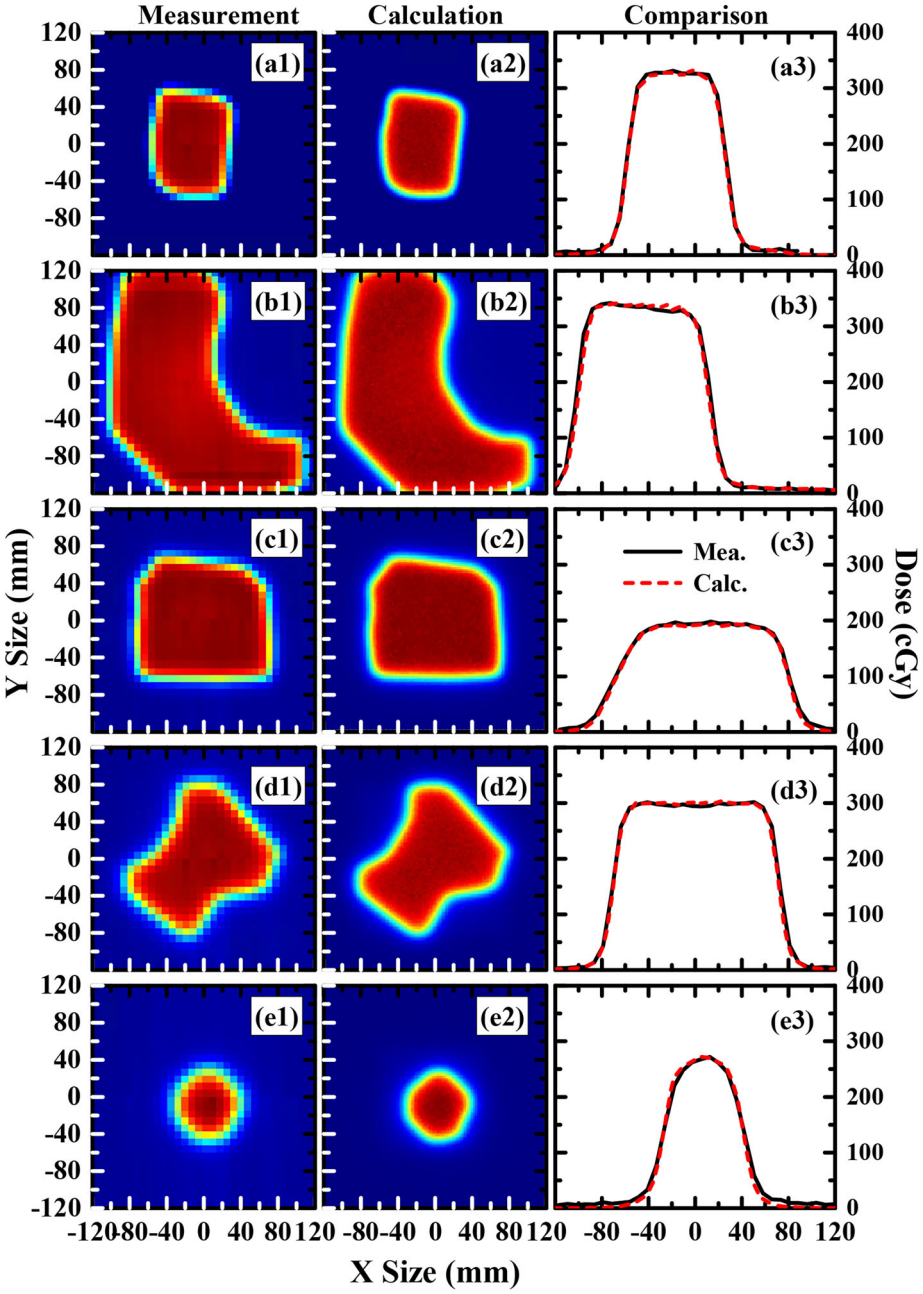
The actual TPS planned dose distribution alongside the measured dose distribution with Matrixx for five clinical cases. (a)–(e) represent case1–case5. The profiles indicate the measured and calculated curves at *Y* = 0 along the *X* direction.

After comprehensive basic validations, we transplanted five previously treated clinical cases from other treatment planning systems into uTPS, and created patient specific cutouts suitable for the VisionaryTx linac to verify the accuracy of the uTPS eMC algorithm in handling actual clinical cases. We measured the point and plane doses of these five clinical cases using CC13 and MatriXX, respectively, and then compared them with the calculated results. The point doses were measured on the central axis of the linac. The energies, SSDs and comparison results of these cases are shown in Table 10. Compared with the previous three cutout shapes, that is, 5 cm circle, 3× 12 ${\mathrm{cm}}^{2}$ diagonal, and L‐Shape, these patient specific cutouts appear more irregular. Figure 6 shows the actual TPS planned dose distribution alongside the measured dose distribution with MatriXX for five clinical cases. The profiles indicate the measured and calculated curves at *Y* = 0 along the *X* direction.

**TABLE 10:** The results of plane and point doses verification with patient specific cutout. The gamma criteria of 3mm, 3% and 10% dose threshold was used.

No.	Energy	SSD (cm)	Depth (cm)	Passing rates	Mea. (cGy)	Cal. (cGy)	Diff.
Case 1	9 MeV	110	2	98.1%	269.3	267.37	0.7%
Case 2	6 MeV	100	1.3	99.6%	327.7	325.1	0.8%
Case 3	6 MeV	100	1.3	96.5%	318.6	312.21	2.0%
Case 4	6 MeV	100	1.3	100.0%	297.7	301.97	−1.4%
Case 5	12 MeV	107	2.6	99.1%	193.6	189.74	2.0%

As shown in Table 10, for plane dose, with the criteria of 3mm, 3% and 10% dose threshold, the lowest passing rate was 96.5% and the highest was 100%. For point dose, dose deviations were within 2%. As can be seen in Figure 6, the measured profiles along the *X* direction at *Y* = 0 are well reproduced by the calculations, not only the absolute doses but also the penumbras.

### 3.7 Evaluation of dose calculation time

**FIGURE 7:**
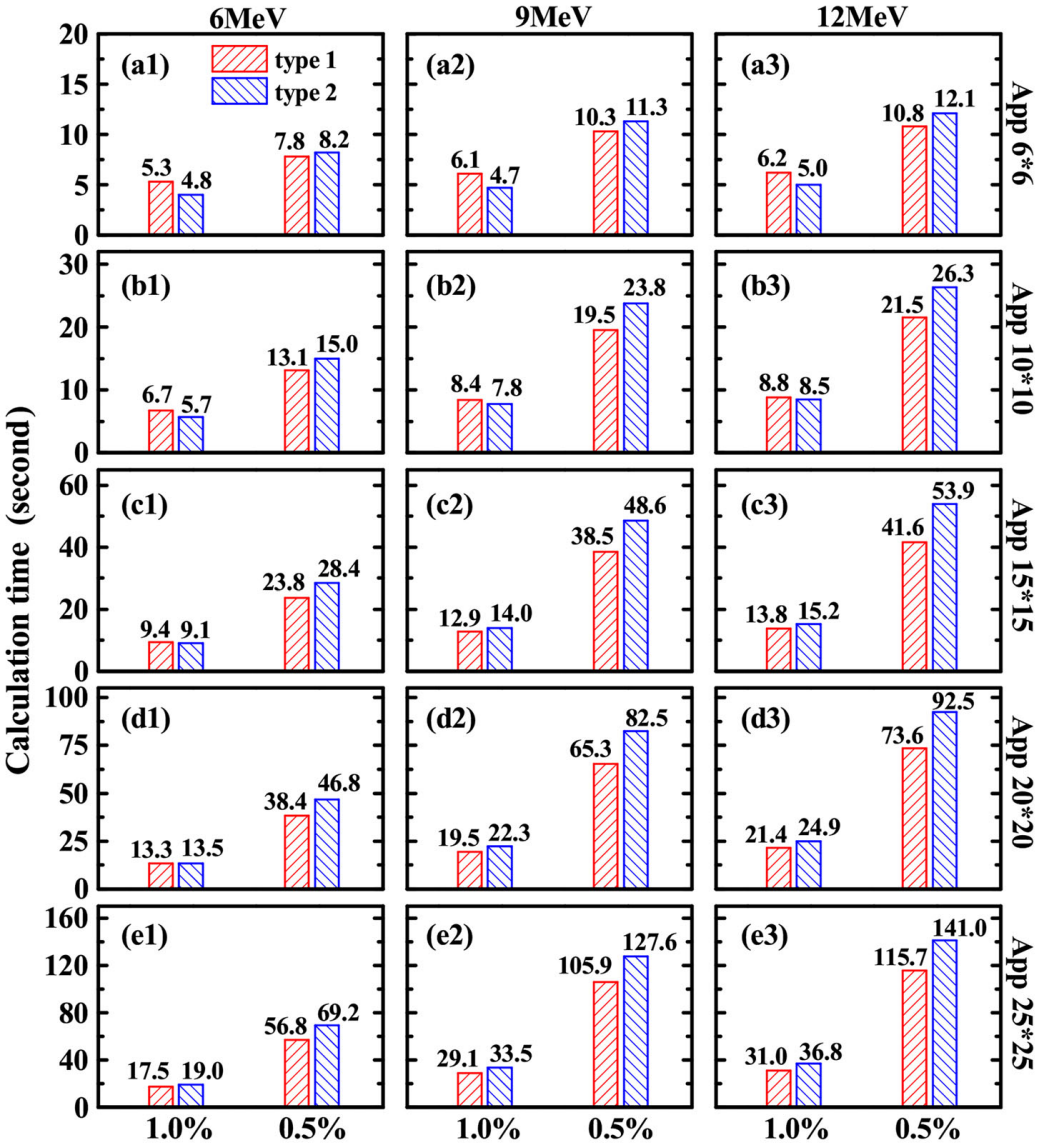
The calculation time of eMC algorithm for different applicators with calculation uncertainties as 1.0% and 0.5% for 6 MeV, 9 MeV, and 12 MeV, in the unit of second.

The calculation speed is another side of the algorithm performance. Several factors affect the calculation time of the MC calculation, including calculation uncertainty, dose grid resolution, and calculation hardware. To evaluate the calculation speed more comprehensively, in the present study, the calculations were performed in two different calculation workstations with the calculation uncertainties set as 1% and 0.5%, respectively. The dose grid resolutions were all set as 2 mm. In the calculations, the corresponding number of particles used in the MC algorithm ranges from about 1.6×10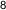 of 6 MeV with 6×6 ${\mathrm{cm}}^{2}$ applicator to about 4.5×10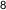 of 12 MeV with 25×25 ${\mathrm{cm}}^{2}$ applicator under 1% uncertainty. The resulting calculation times are illustrated in Figure 7.

As displayed in Figure 7, with the increase of the energy and applicator size, the calculations become longer. For the calculations with 1% uncertainty, nearly all the calculation times are within 30 s except the calculations of 12 MeV with applicator 25×25 ${\mathrm{cm}}^{2}$, which are 31 s and 36.8 s for type 1 and type 2 workstations, respectively. While for those with 0.5% uncertainty, the maximum calculation time is nearly 2.5 min. The calculation times of 0.5% are about 1.4 – 3.8 times of those of 1%.

## 4 DISCUSSION

### 4.1 Dose calculation accuracy

In Tables 4, 5, and 6, a total of 405 point doses were measured and compared with the calculations for normal and extended SSDs. For SSD 100 cm, the average absolute dose difference for 6 MeV, 9 MeV, and 12 MeV are 1.03%, 0.71%, and 0.70%, respectively. For SSD 105 cm, the average absolute dose difference for 6 MeV, 9 MeV, and 12 MeV are 2.46%, 1.76%, and 1.55%, respectively. For SSD 110 cm, the average absolute dose difference for 6 MeV, 9 MeV, and 12 MeV are 2.95%, 2.01%, and 1.10%, respectively. The results for 6 MeV were slightly larger than those of 9 MeV and 12 MeV. This may be due to the reason that there are more low energy scatter particles from the 6 MeV measurement than those from 9 MeV and 12 MeV. The behaviors of these low energy scatterings are more difficult to simulate accurately than high energy.^31^, ^32^ With increasing SSDs, the absolute dose differences were generally larger. For most cases, the dose differences at the deepest position were larger than other position, this is because the absolute dose at these position are smaller, and small dose differences would result in large relative dose differences.

As required in IAEA 1540,^33^ the dose differences for photon beam in the homogeneous phantom with simple square and rectangular fields are within ±2% for central axis data and ±3% for off‐axis data. Since the measurement uncertainty of electron beams is larger than that of photon beams,^34^, ^35^ therefore, the dose difference shown in Tables 4, 5, and 6 are quite good for electron beams. According to the IAEA TRS 398^34^ as well as AAPM TG‐51 protocols^36^ and its addendum,^37^ during the point dose measurements using the CC13 ion chamber with 6, 9, and 12 MeV electron beams, the measurement uncertainty including the combined standard uncertainty $u_{c}$ and the expanded uncertainty U could be calculated using the following formula:

(3) $$u_{c}=\sqrt{u_{A}+u_{B,total}},U=2*u_{c}\left(k=2\right)$$

The $u_{A}$ uncertainty 0.10% was derived from repeated measurements, while $u_{B,\text{total}}$ components were adopted from TG‐51 and TRS‐398, including the uncertainties of calibration factor *N* 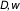, beam quality correction factor $k_{Q}$, and temperature and pressure correction $k_{T,P}$ etc. Finally, the combined standard uncertainties were about 0.99% for 6 MeV, 0.75% for 9 MeV, and 0.72% for 12 MeV, respectively. The expanded uncertainties (*k* = 2) were about 1.98% for 6 MeV, 1.50% for 9 MeV, and 1.44% for 12 MeV.

In the plane dose verifications, the reason for poorer GPRs of 25×25 ${\mathrm{cm}}^{2}$ applicator is because the detector area of MatriXX is around 24×24 ${\mathrm{cm}}^{2}$, which is smaller than the maximum applicator size and result in the dose difference around the detector edge of MatriXX. The GPRs of extended SSD, that is, SSD equal to 105 cm and 110 cm, show no big difference from those of SSD equal to 100 cm, indicating that the eMC could simulate the electron scattering with different air gap between applicator and patient/phantom properly.

However, it should be mentioned that, as suggested in MatriXX's user manual, the calculation was performed on a virtual phantom, for which the relative electron density was set to 1.016, as recommended by the manufacturer for RW3 material. The dose at the same positions as the measurements was then extracted and compared with the measurement results. However, unlike using a virtual phantom, in actual measurements, the MatriXX device introduces the following differences. First, the MatriXX detector is an ion chamber array, and its housing, circuit board, and detection units differ from homogeneous virtual phantom in effective atomic number and electron density. Replacing part of the setup with a virtual phantom alters the attenuation, scattering characteristics, and energy deposition process in the local radiation field, leading to discrepancies between the direct detector measurements and the dose in a virtual phantom at the same location. Furthermore, in regions with large dose gradients, the volume‐averaging effect of the detector itself further amplifies the measurement deviations. As mentioned in Ref 38, the MatriXX exhibits angular dependence under oblique incidence, especially at angles of 91
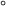
–110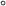 and 260
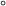
–269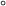. However, the results also indicate that the angular dependence is not significant at 30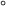,^38^ so we did not apply angular dependence corrections to the oblique‐incidence MatriXX measurement data. Applying such angular corrections would improve the passing rates for oblique cases.

For the calculations with cutout, a cutout transmission factor need to be input. Refer to AAPM TG106,^39^ the cutout transmission factor is the ratio of the point dose with and without the cutout measured by CC13 ion chamber with the 10×10 applicator at the respective *d* 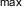 depths of SSD 100 cm. In the present study, the transmission factors were all set as 0.02.

The verification results for the heterogeneous phantom meet the requirement in AAPM MPPG 5.b that the difference between measurement and calculation is less than 7%, demonstrating the high accuracy of the uTPS MC algorithm in heterogeneous media. The results with cutouts, both special designed and patient specific, indicate that the eMC algorithm could treat the scenario with cutout properly. Therefore, through the comparisons from simple to complicate cases, the dose calculation accuracies for both normal and extended SSD meet the requirements of clinical use for electron radiation mode with and without cutout. Validation with clinical cases demonstrates that the eMC algorithm of uTPS also achieves good accuracy in handling practical clinical scenarios.

In reference,^12^ an eMC model in Monaco that already approved for clinical use have been used to the calculation in both homogeneous and heterogeneous phantoms. In a homogeneous phantom, the point dose deviations of the TPS were ≤4%. In heterogeneous phantom, energies above 6 MeV show better than 4% agreement between calculation and measurement. In a validation of the eMC algorithm in RayStation,^13^ the AAPM MPPG 5.a guideline was followed to verify the algorithm accuracy under various conditions, including square cutouts, custom cutouts, oblique incidence, and heterogeneous media. Overall, excellent agreement was observed between ion chamber measurements and RayStation calculations. Among the 312 output factors for square cutouts, 6.4% exceeded 3% and 1.6% exceeded 5%. In the study,^10^ the accuracy of the Eclipse eMC algorithm was verified using MatriXX. The results show that Gamma analysis at 3%/3mm showed very good agreement (>95%) for all cases. Previous studies indicate that the eMC algorithm of uTPS demonstrates accuracy comparable to those of other commercial treatment planning systems.

### 4.2 Dose calculation speed

As mentioned in AAPM TG105^28^ and AAPM TG157,^40^ proper use of variance reduction techniques enables MC algorithms to meet clinical requirements for computational efficiency. However, improper use of variance reduction techniques may lead to unpredictable results. The variance reduction techniques employed are described in both RayStation and Monaco manuals. In RayStation, a range of common MC variance reduction methods are used to enhance the efficiency of particle transport. Particles that impact the edge, top, or front face and do not experience out‐scattering are discarded, and compensated by splitting some of the remaining particles. The statistical weight is modified such that the total fluence remains unchanged, and the split particles are transported separately afterward. Furthermore, the Russian roulette technique is applied to lower the population of secondary low‐energy electrons in the beam line transport. In Monaco, variance reduction techniques such as interaction forcing, electron history repetition and Russian roulette are used in the simulation of photon histories. The Macro Monte Carlo (MMC) method^41^ is used in Eclipse to transports electrons and calculates the dose deposited along the particle trajectories. The calculated dose distribution can be smoothed to reduce the statistical noise and the amount of time spent on the calculation. The purpose of smoothing is to preserve meaningful structures in the dose distribution while smoothing out the noise. For Eclipse, the eMC computation time ranged from 5 to 66 min on a single 2.66 GHz desktop.^7^ When using a CPU with 16 processors (Intel Xeon E5‐2690, 2.9 GHz), the dose calculation times with 1% statistical uncertainty reduce to 30 s and 24 s, for the retromolar trigone and nose treatment plans, respectively.^9^

In the MC algorithm of uTPS, variance reduction techniques such as particle splitting, Russian roulette, electron history repetition, and smoothing method were not employed. However, to ensure computational efficiency, in addition to the asynchronous CPU+GPU architecture mentioned above, the uTPS MC algorithm has been extensively optimized for GPU parallel computing, including optimizations in thread allocation, GPU memory usage optimization, and reduce thread divergence etc. In uTPS, the GPU parallel calculation technique is used for all dose calculation algorithms, including CC, MC, and eMC. With the help of GPU acceleration technology, the calculation time could be significantly reduced, meeting clinical usage requirements. Moreover, it should be mentioned that in order to keep the dose calculation accuracy, no variance reduction techniques are used in the (electron) MC algorithm of uTPS.

As shown in Figure7, through the comparisons of the calculation time from the two different calculation workstations, that is, type1 and type2, it could be seen that the tendencies of different calculation workstations and calculation uncertainties for each energy are similar. Let's take the results of 6 MeV as an example. It can be seen that for the calculations with 1% uncertainty, the calculation time from type1 workstation with 6×6 ${\mathrm{cm}}^{2}$, 10×10 ${\mathrm{cm}}^{2}$ and 15×15 ${\mathrm{cm}}^{2}$ applicators are less than those from the type2 workstation. The situation is opposite for the calculations with 20×20 ${\mathrm{cm}}^{2}$, 25×25 ${\mathrm{cm}}^{2}$ applicators. This can be explained by the following reasons. The electron dose calculations can be separated into two parts. The first part is data preparation, including the calculation of the fluence map, the handling the CT image etc., and is performed on the CPU. The second part is the sampling and transport of the primary and secondary particles, and is performed on the GPU. The less advanced CPUs in type1 workstation required more time to prepare the input data than type2. For the case with less particle numbers, the advantage of more advanced GPU in type1 workstation becomes not that obvious. The more particle number is the more obvious advantage of the advanced GPU. Therefore, as can be seen from the calculation with 0.5% uncertainty, the calculation time from type1 workstation are much less than those from type2 workstation. When the particle number was sufficient enough, the calculation times with 0.5% uncertainly were almost four times of those with 1% uncertainty, for example the result of 25×25 ${\mathrm{cm}}^{2}$ applicator with 12 MeV. This tendency satisfies the inverse relationship between the square of uncertainty and particle number. Clinically, the calculation uncertainty of the eMC algorithm is usually set as 1%. Therefore, the maximum calculation time with a 2 mm grid spacing was within 40 s. This definitely meets the requirement of calculation time in the clinical use. These results also indicate that, if more advanced hardware is used, the calculation time could be further reduced.

## 5 CONCLUSIONS

This paper presents the commissioning and performance evaluation of the MC based electron dose calculation algorithm in uTPS. The electron beam data of 6 MeV, 9 MeV, and 12 MeV with all applicators from the newly developed dual‐layer MLC CT‐linac uLinac VisionaryTx were modeled. The agreement between the measured and calculated beam data was quite good through direct comparison and one dimensional gamma and DTA passing rates. In the dose calculation accuracies evaluation, the AAPM MPPG 5.b and other AAPM guidelines were followed, the detailed comparisons between calculations and measurements were performed, including normal and extended SSD, oblique incidence, cutouts, heterogeneous phantoms, and clinical cases. Verifications performed under these basic and complicated conditions indicate that the accuracy of eMC algorithm in uTPS met the requirements of MPPG 5.b for electron beam dose verification. The eMC algorithm of uTPS demonstrates accuracy comparable to that of other commercial TPSs. In the evaluation of calculation speed, the maximum calculation times were within 40 and 150 s for the calculation uncertainty of 1% and 0.5%, respectively. Therefore, through the performance evaluations in the present study, we may conclude that both the dose calculation accuracy and calculation speed of the eMC algorithm in uTPS met clinical usage requirements.

## AUTHOR CONTRIBUTIONS

Acquisition of data: Feng Wang, Xuming Jiang and Zhi Shi. Analysis and interpretation of data:Feng Wang, Xuming Jiang, Zhi Shi and Yanfang Liu. Drafting of the manuscript: Feng Wang. Supervision of the project, overall direction and planning: Jiayi Chen and Yibin Zhang. Final approval of manuscript: All authors.

## CONFLICT OF INTEREST STATEMENT

The authors declare no conflicts of interests.

## FUNDING INFORMATION

This work was supported partially by the National Natural Science Foundation of China(Grant No.: 82373514), Noncommunicable Chronic Diseases‐National Science and Technology Major Project (Grant No.: 2023ZD0502200) and Shanghai Innovative Medical Device Application Demonstration Project(Grant No.: 23SHS03400).

## ETHICS STATEMENT

This study was approved by Shanghai Ruijin Clinical Trial Ethics Committee ‐ approval: (2023)EAN(118)‐2. The study's clinical trial registration number is ChiCTR2400082631 registered with Clinical trial to evaluate the safety and efficacy of medical electron linear accelerator for tumor radiation therapy.
